# Realizing Sunlight‐Induced Efficiently Dynamic Infrared Emissivity Modulation Based on Aluminum‐Doped zinc Oxide Nanocrystals

**DOI:** 10.1002/advs.202405962

**Published:** 2024-07-29

**Authors:** Yan Jia, Dongqing Liu, Desui Chen, Yizheng Jin, Yufei Ge, Wenxia Zhang, Chen Chen, Baizhang Cheng, Xinfei Wang, Tianwen Liu, Mingyang Li, Mei Zu, Zi Wang, Haifeng Cheng

**Affiliations:** ^1^ Science and Technology on Advanced Ceramic Fibers and Composites Laboratory College of Aerospace Science and Engineering National University of Defense Technology Changsha 410073 P. R. China; ^2^ Center for Chemistry of High‐Performance and Novel Materials State Key Laboratory of Silicon Materials Department of Chemistry Zhejiang University Hangzhou 310027 China

**Keywords:** infrared emissivity, localized surface plasmon resonance, photo‐induced

## Abstract

Dynamic manipulation of an object's infrared radiation characteristics is a burgeoning technology with significant implications for energy and information fields. However, exploring efficient stimulus–spectral response mechanism and realizing simple device structures remains a formidable challenge. Here, a novel dynamic infrared emissivity regulation mechanism is proposed by controlling the localized surface plasmon resonance absorption of aluminum‐doped zinc oxide (AZO) nanocrystals through ultraviolet photocharging/oxidative discharging. A straightforward device architecture that integrates an AZO nanocrystal film with an infrared reflective layer and a substrate, functioning as a photo‐induced dynamic infrared emissivity modulator, which can be triggered by weak ultraviolet light in sunlight, is engineered. The modulator exhibits emissivity regulation amount of 0.72 and 0.61 in the 3–5 and 8–13 µm ranges, respectively. Furthermore, the modulator demonstrates efficient light triggering characteristic, broad spectral range, angular‐independent emissivity, and long cyclic lifespan. The modulator allows for self‐adaptive daytime radiative cooling and nighttime heating depending on the ultraviolet light in sunlight and O_2_ in air, thereby achieving smart thermal management for buildings with zero‐energy expenditure. Moreover, the potential applications of this modulator can extend to rewritable infrared displays and deceptive infrared camouflage.

## Graphical abstract

A novel efficient dynamic infrared emissivity regulation mechanism and a structurally simple photo‐induced dynamic infrared emissivity modulator is developed. The modulator exhibits efficient stimulus–spectral response performance, a large emissivity modulation amount, and adaptive emissivity conversion characteristics depend on ultraviolet and oxygen, demonstrating potential applications in dynamic radiative cooling, infrared adaptive camouflage, and rewritable infrared display.

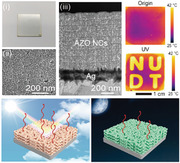

## 1 Introduction

Although infrared radiation is imperceptible to the human eye, all objects in our surroundings emit infrared radiation continually. Dynamic infrared emissivity regulation (DIE) technology is a flexible, portable, and energy‐efficient technique to manipulating infrared radiation, which is of vital importance to a broad range of areas, from smart thermal management to adaptive infrared camouflage and dynamic information display.^[^ ^1^, ^2^, ^3^, ^4^ ^]^ For instance, the temperature of buildings or the human body can be altered by dynamic regulating their own emissivity, thereby changing the radiative heat exchange between themselves and the environment, achieving radiative cooling and heating. In military camouflage, the infrared exposure signature of military targets can be proactively integrated into the background environment by actively modulating their own infrared emissivity, thereby countering infrared reconnaissance. However, the development of DIE technology has proven to be a nontrivial challenge because several rigorous technical requirements need to be simultaneously satisfied: simple triggering mode, reversible regulation, large regulation amounts, wide spectral regulation range, and highly robust device structure.^[^ ^5^ ^]^

To date, only a limited number of DIE systems have been reported, categorized by the mode of triggering, including electricity,^[^ ^3^, ^6^, ^7^, ^8^ ^]^ thermal,^[^ ^9^, ^10^, ^11^ ^]^ force,^[^ ^12^, ^13^, ^14^, ^15^, ^16^, ^17^, ^18^, ^19^ ^]^ light,^[^ ^20^, ^21^, ^22^, ^23^, ^24^, ^25^, ^26^ ^]^ and others. In these modes, light triggering mode is distinguished by non‐contact, remote control, and high temporal and spatial resolution. Whatmore, the photo‐induced DIE technology offers several advantages: 1) simple structure; 2) pixel‐level emissivity modulation by controlling the illumination source or applying an intermediate masking layer; 3) fast response speed; 4) multistate modulation of emissivity, etc. The few existing photo‐induced DIE mechanisms are predominantly based on the modulation of optical loss in metamaterials^[^ ^20^ ^]^ and the laser‐induced thermal phase change of materials (e.g., GeSbTe^[^ ^21^, ^22^, ^23^, ^24^, ^25^ ^]^ and VO_2_^[^ ^26^ ^]^). However, the former's metamaterial structures are complex to fabricate, and the emissivity modulation amount is relatively small. And the latter requires a higher energy consumption for laser heating, leading to a low stimulus–spectral response conversion efficiency.

Here, we developed a photo‐induced dynamic infrared emissivity (PDIE) modulator, comprised with a bearing substrate and a simple double‐layer film architecture. The upper layer of this architecture is an aluminum‐doped zinc oxide (AZO) nanocrystal film, serving as the stimulus‐responsive layer, while the lower layer is comprised of either an Ag film or an indium tin oxide (ITO) film, functioning as the infrared reflective layer. The emissivity modulation is achieved by regulating the infrared localized surface plasmon resonance (LSPR) absorption intensity of AZO NCs through photocharging/oxidative discharging. The photosensitivity of AZO NCs enables even weak ultraviolet (UV) light in sunlight to induce emissivity regulation of the PDIE modulator. We demonstrate that PDIE modulator is capable of self‐adaptive switching their emissivity states in response to UV light in sunlight and O_2_ in air, with zero energy consumption. This performance renders the PDIE modulator suitable for applications in building rooftops and smart windows, thereby contributing to the reduction of building energy consumption.

## 2 Results and Discussion

### 2.1 Preparation and Working Principle

**Figure 1:**
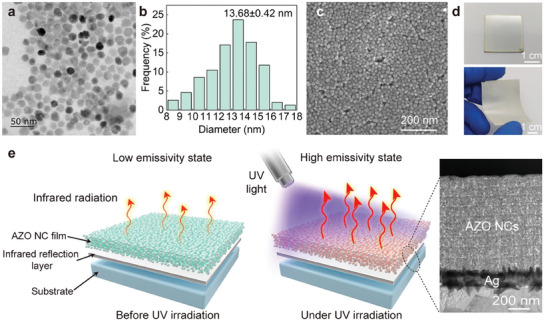
Morphologies and working principle of the PDIE modulator. a) TEM image of AZO NCs. b) Size distribution of AZO NCs. c) Surface morphologies of the PDIE modulator. d) Rigid and flexible PDIE modulators using Al sheets and polyethylene glycol terephthalate films as substrates, respectively. e) Working principle of the PDIE modulator. Inset: the cross‐sectional morphologies of the PDIE modulator.

We first synthesized spheroidal and pyramidal AZO NCs with a diameter of 13.68 ± 0.42 nm and Al doping amount of 0.96%, shown in **Figure** **1**a,b. AZO NCs exhibit the wurtzite structure characteristic of ZnO (Figure S1, Supporting Information). We prepared AZO NC film by spin‐coating process, which is formed by the tightly packed AZO NCs bonded through van der Waals forces (Figure 1c).^[^ ^27^ ^]^ The AZO NC film exhibited the LSPR absorption peak with the peak position located at 7.09 µm (Figure S2, Supporting Information). The peak width of LSPR covers the entire infrared atmospheric window owing to free carrier motion scattering.^[^ ^28^ ^]^ Therefore, LSPR regulation of the AZO NC film can be achieved at mid‐wave infrared (MWIR; 3–5 µm) and long‐wave infrared (LWIR; 8–13 µm), where objects radiate heat outward through these infrared atmospheric windows. The LSPR regulation of AZO NC film involves the regulation of absorbance and transmittance, and an infrared‐reflective background is needed to convert infrared transmittance to infrared reflectivity. In this way, the PDIE modulator can transition between the high infrared absorption state (high emissivity state) and the high infrared reflection state (low emissivity state). According to the Hagen–Rubens relation, $R=1-2\sqrt{\frac{2\varepsilon_{0}\omega }{\sigma }}$, where *R* is reflectivity, *ε*_0_ is the vacuum permittivity, *ω* represents the frequency of infrared light, *σ* is the conductivity,^[^ ^29^ ^]^ films with high conductivity typically exhibit a strong shielding and reflection effect on infrared radiation. Therefore, Ag or ITO films with high conductivity are selected as the infrared reflection layer in this paper. The PDIE modulator can be prepared on flexible large‐area (such as polymers) and rigid (including glass, metal, and others) substrates (Figure 1d).

Using Ag film as the infrared reflection layer and Al sheet as the substrate, we prepared the PDIE modulator (Inset of Figure 1e). The Ag film is set at 135 nm, and at this thickness, the Ag film can provide an effective infrared reflection background (reflectivity: 97.7% in 3–13 µm). The UV light can photodope the AZO NCs, changing their carrier concentration and regulating their LSPR absorption intensity. As shown in Figure 1e, in the initial state, the LSPR absorption of AZO NCs is weak while infrared transmittance is strong. The PDIE modulator exhibits the high infrared reflectivity characteristics of the underlying Ag film. When exposed to UV irradiation, the carrier concentration of AZO NCs increases resulting in the increase of LSPR absorption intensity. Therefore, the PDIE modulator exhibits the high emissivity state. Upon cessation of UV exposure, the photogenerated carriers of AZO NCs are gradually oxidized by O_2_, resulting in a weakening of the LSPR absorption and an increase in infrared transmittance. Consequently, the PDIE modulator reverts to the low emissivity state.

### 2.2 Regulation Performance

**Figure 2:**
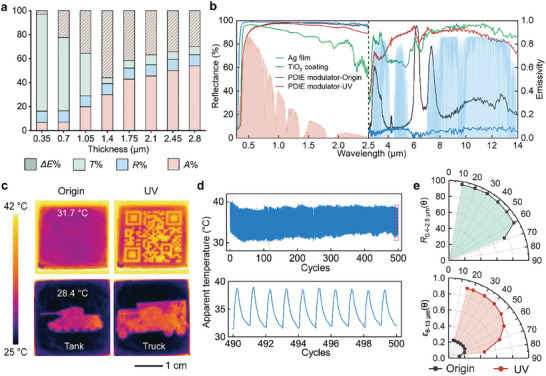
Performance of the PDIE modulator. a) The ratio of infrared emissivity regulation (Δ*E*%), infrared transmittance (*T*%), infrared reflectance (*R*%), and infrared absorbance (*A*%) of AZO NC film as a function of film thickness. b) UV–vis–NIR reflection spectra and infrared emissivity spectra of a TiO_2_ coating (green), Ag film (blue), and PDIE modulator in the initial state (black) and under exposure to UV irradiation (red). Normalized ASTM G173 Global solar spectrum (pink) and transparent infrared atmospheric window (blue) are plotted for reference. c) Infrared thermal images of the PDIE modulator before and after exposure to UV irradiation. The heat plate temperature is 40 °C. d) The apparent temperature change of the PDIE modulator as the number of cycles increases. The heat plate temperature is 40 °C. e) Average angular reflectivity at 0.4–2.5 µm (*R*_0.4–2.5 µm_(𝜃)) and average angular emissivity at 8–13 µm (*ε*_8–13 µm_(𝜃)) of the PDIE modulator. Angular reflectivity is measured by specular angle.

The infrared emissivity regulation performance of PDIE modulators is mainly influenced by the film thickness of AZO NC film. To explore the potential emissivity modulation performance of the PDIE modulator, we studied the spectral characteristics of the AZO NC films as a function of film thickness (**Figure** **2**a). For AZO NC films with thicknesses less than 1.4 µm, the LSPR absorption (*A*%) enhances and the emissivity regulation amount (Δ*E*%) gradually increases as the film thickness increases. However, for AZO NC films thicker than 1.4 µm, the infrared transmittance (*T*%) used to regulate emissivity decreases with increasing *A*%, resulting in a reduction in Δ*E*%. These trade‐offs imply that ≈1.4 µm is the optimal AZO NC film thickness for achieving maximum Δ*E*%.

Figure 2b shows the spectral characteristics of the optimized PDIE modulator compared to Ag film and commercial TiO_2_ radiative cooling coating. The diffusive component of reflectivity and emissivity is integrated during this measurement. The PDIE modulator exhibits high reflectivity of 94.2% in the visible and near‐IR (vis–NIR; 0.4–2.5 µm) bands. This is primarily attributed to the high transmittance of AZO NC films (>93%; Figure S3, Supporting Information) and the high reflectivity of Ag films (>98%; Figure 2b) within this band. Compared with the low emissivity state of the Ag film, the infrared emissivity of the PDIE modulator can be regulated between the high‐emissivity state (0.83 in MWIR, 0.89 in LWIR; Note S1, Supporting Information) and the low‐emissivity state (0.11 in MWIR, 0.28 in LWIR), due to the emissivity regulation function of AZO NC films. The PDIE modulator under UV irradiation has higher vis–NIR reflectivity and comparable infrared emissivity than commercial TiO_2_ radiative cooling coatings (Figure 2b), allowing it to exhibit similar radiative cooling performance to TiO_2_ coatings. The emissivity regulation amount of PDIE modulator is 0.72 and 0.61 in MWIR and LWIR, respectively. Compared to other photo‐induced emissivity modulators,^[^ ^20^, ^21^, ^22^, ^23^, ^24^, ^25^, ^26^ ^]^ the PDIE modulator not only exhibits a superior emissivity modulation amount but also features a more straightforward fabrication process (Table S1, Supporting Information). In addition, PDIE modulator can realize an infrared radiation power modulation of 87.9 W m^−2^ in the LWIR at 298 K (Note S2, Supporting Information).

By applying different UV masks, the PDIE modulator can achieve high‐resolution infrared pattern display. As shown in Figure 2c, the PDIE modulator displays the pattern of QR code, which can be utilized for rewritable infrared information encryption. The PDIE modulator can also realize different infrared signature displays, such as the infrared signature of tanks and trucks, which can be used for nighttime infrared camouflage and deceptive infrared targets. The cycling stability is a key indicator for infrared emissivity dynamic modulation devices. The PDIE modulators were able to be stably cycled over 500 times and maintain a high apparent temperature change of 7.3 °C (Figure 2d). This high durability is attributed to the photocharging/oxidative discharging process, which does not cause structural damage to the AZO NCs, thereby ensuring the long‐term stable operation of the PDIE modulator.

Angular reflectivity and angular emissivity properties of PDIE modulator are crucial for the application of PDIE modulators in radiative cooling fields. Angular reflectivity and angular emissivity were measured by specular angle. The PDIE modulator exhibits high Vis‐NIR reflectance (>93%) for solar zenith angles of 10°–60°, while this reflectance decreases to 75.1% at the maximum tested angle of 68° (Figure 2e and Figure S4a, Supporting Information). Before UV irradiation, the PDIE modulator exhibits low infrared emissivity (<0.24) from 15° to 80° (Figure 2e and Figure S4b, Supporting Information). During UV irradiation, the PDIE modulator shows high infrared emissivity (>0.88) from 15° to 40°. The infrared emissivity gradually decreases from 0.86 to 0.46 as the zenith angle gradually increases from 50° to 80°. At large zenith angles, i.e., close to horizontal, the lower infrared emissivity is conducive to blocking radiative heat transfer to the PDIE modulator by incoming atmospheric radiation,^[^ ^30^ ^]^ which is beneficial for daytime radiative cooling of PDIE modulators.

### 2.3 Regulation Mechanism

**Figure 3:**
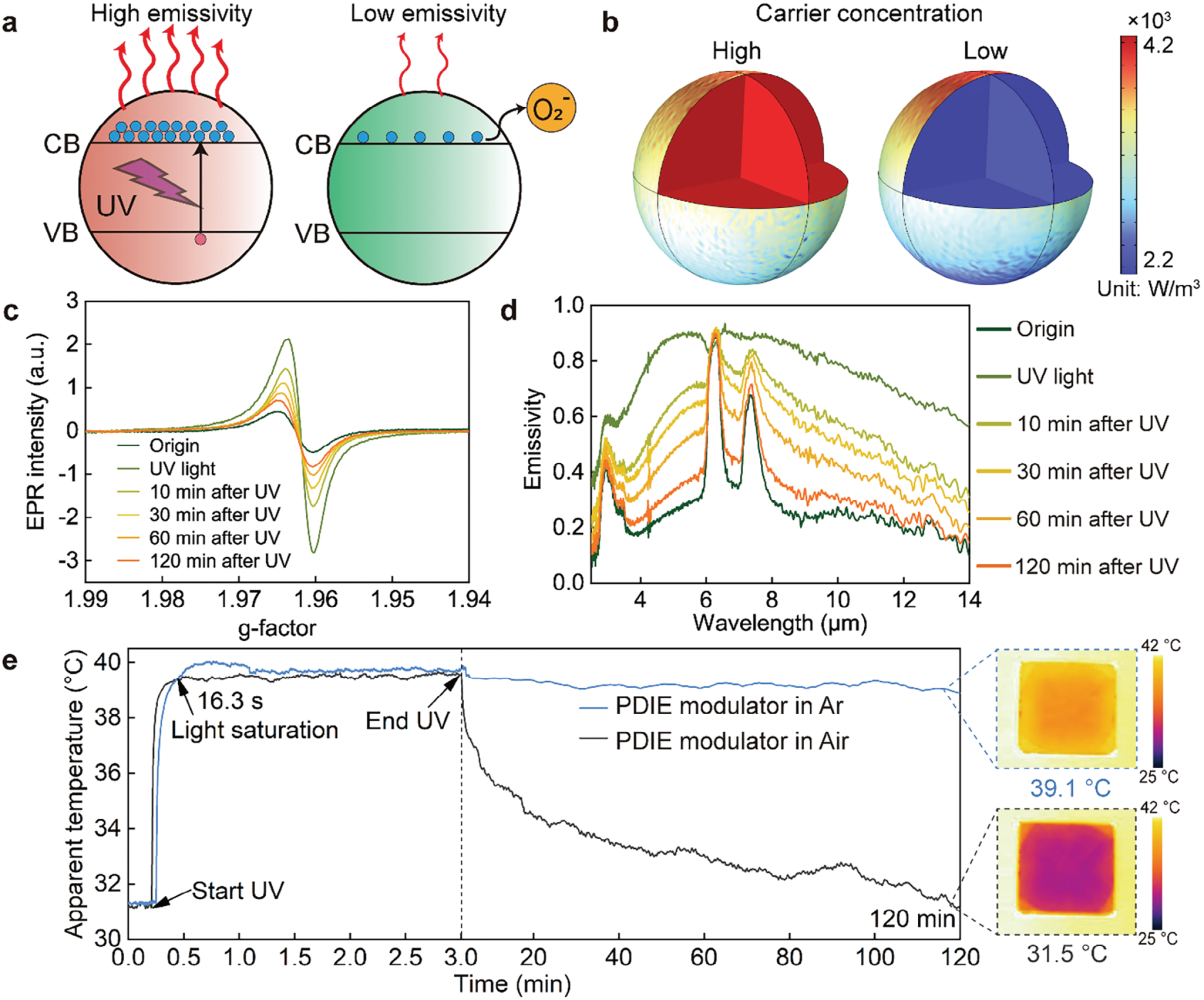
Emissivity regulation mechanism of PDIE modulators. a) Schematic of high‐emissivity and low‐emissivity states of AZO NCs under UV light excitation and O_2_ exposure after UV irradiation, respectively. VB: valence band; CB: conduction band. b) Simulated electromagnetic power loss density of AZO NC at high and low electron carrier concentration. c) EPR spectra of AZO NCs in toluene before, under, and after UV irradiation. d) Infrared emissivity regulation of the PDIE modulator before, under, and after UV irradiation. e) Apparent temperature change of the PDIE modulator in air and Ar environments before, under, and after UV irradiation.

**Figure** **3**a shows a schematic diagram of photocharging/oxidative discharging of AZO NCs. The UV irradiation can excite electrons in the valence band into the conduction band, leading to the accumulation of conduction band electrons and an increase of carrier concentration in AZO NCs.^[^ ^20^, ^31^, ^32^, ^33^, ^34^ ^]^ When the UV illumination is stopped, O_2_ captures light‐excited free carriers, and the carrier concentration of the AZO NCs decreases.^[^ ^20^, ^31^, ^32^, ^33^, ^34^ ^]^ Thus, the carrier concentration of AZO NCs can be regulated through photocharging/oxidative discharging. We further simulated the electromagnetic power loss density of AZO NCs at high and low carrier concentrations to represent the changes in the LSPR absorption intensity of the AZO NCs (Note S3, Supporting Information). At high carrier concentrations, the electromagnetic power loss density is high and the LSPR absorption is strong; conversely, at low carrier concentrations, the LSPR absorption is weak (Figure 3b). The LSPR absorption intensity of doped oxide NCs can be dynamically modulated by regulating the carrier concentration of NCs, which has been reported in electrochromic films based on doped oxide NCs (by electrically regulating the carrier concentration).^[^ ^28^, ^35^, ^36^, ^37^ ^]^ Therefore, the infrared LSPR of AZO NCs can be regulated through photocharging/oxidative discharging, so as to realize the modulation of the infrared emissivity of AZO NCs (Figure 3a).

To obtain further insight into the infrared emissivity regulation mechanism of the PDIE modulator induced by UV photocharging/oxidative discharging, in situ electron paramagnetic resonance (EPR) and Fourier transform infrared spectroscopy (FTIR) studies have been carried out. Figure 3c shows the EPR spectra of the AZO NCs collected before, during, and after UV irradiation. The EPR peak signal at *g* = 1.962 is attributed to the conduction band electrons driving from lattice electron trapping sites.^[^ ^38^, ^39^ ^]^ Increasing the number of conduction band electrons in the AZO NCs leads to an increase in EPR intensity.^[^ ^31^ ^]^ When the AZO NCs are in situ irradiated by UV light, the EPR signal of the AZO NCs enhances, indicating that an increase in the number of conduction band electrons in AZO NCs induced by photocharging (Figure 3c). This leads to a corresponding enhancement in LSPR absorption of AZO NCs and results in a high infrared emissivity state for the PDIE modulator (Figure 3d). Upon cessation of UV irradiation, the photoexcited electrons undergo gradual oxidation, resulting in a weakening of the EPR signal (Figure 3c). This leads to a corresponding reduction in LSPR absorption of AZO NCs and results in a high infrared reflectivity (low emissivity) for the PDIE modulator (Figure 3d).

We analyzed the response time of the PDIE modulator during the photocharging/oxidative discharging using an infrared thermal imager (FLIR T1050sc, detection band: 7.5–14 µm), with the results shown in Figure 3e. The AZO NC film undergoes light saturation after ≈16.3 s of UV irradiation. In this state, the photogenerated carriers reach a saturated state, and the apparent temperature of the PDIE modulator cannot increase further, that is, the infrared emissivity does not increase. The oxidative discharge of the AZO NC film exhibits a significantly longer duration (≈120 min) compared to charging, which is in agreement with the decay time of EPR signals. The PDIE modulator exhibited an apparent temperature change of ≈8.2 °C during emissivity regulation (Figure 3e). The actual temperature of the PDIE modulator (measured by thermocouples) caused by UV irradiation increased negligibly (Figure S5, Supporting Information). Therefore, the observed change in the apparent temperature of the PDIE modulator can be attributed to the regulation of its emissivity. We also investigated the apparent temperature changes of an PDIE modulator before and after UV irradiation in an O_2_‐free argon environment in a glove box (Figure 3e). After stopping UV irradiation, the apparent temperature of the PDIE modulator did not significantly decrease, indicating that O_2_ in the air plays a crucial role in the oxidative discharging process of photoexcited electrons.

We also analyzed whether UV light in sunlight can trigger emissivity regulation of PDIE modulators. The photo‐triggering cutoff frequency of AZO NCs is 378 nm (Figure S6, Supporting Information), which is encompassed within the UV spectrum of sunlight. The UV irradiance of sunlight is greater than 1.92 mW cm^−2^ from 8:00 to 17:00 in the daytime (Figure S7, Supporting Information; March 06, 2023, in Changsha, China). We used an LED flashlight (center wavelength: 365 nm; Starshine ZUV12) with an irradiance of 1 mW cm^−2^ to simulate the weak UV light in the sunlight to irradiate the PDIE modulator. The PDIE modulator exhibited obvious apparent temperature changes, as shown in Figure S8 (Supporting Information). This observation suggests that even weak UV light can effectively regulate the infrared emissivity of the PDIE modulator, allowing for sunlight to induce emissivity regulation (Figure S9, Supporting Information).

### 2.4 Application Demonstration

**Figure 4:**
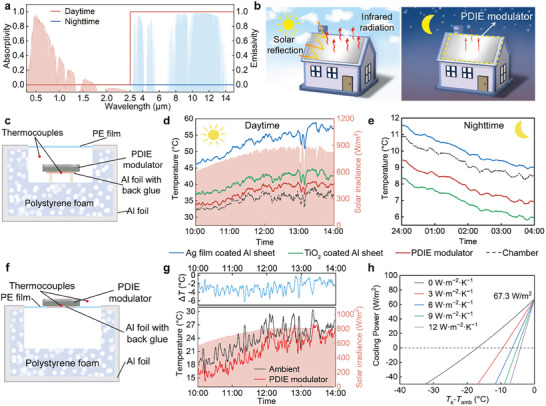
DRC performance of PDIE modulators. a) Absorptivity/emissivity spectrum of ideal dynamic radiation cooling materials. b) Schematic of the DRC principles of the PDIE modulator used on a building roof. c) Schematic of the setup for testing the DRC performance of the PDIE modulators. d) Real‐time solar radiation intensity and temperatures of the PDIE modulator during the daytime (10:00–14:00). e) Real‐time temperatures of the PDIE modulator during the nighttime (0:00–4:00). f) Schematic of the experimental setup used to evaluate the DRC performance of the PDIE modulator in an open environment without a convection shield. g) Real‐time data for the solar radiation intensity and temperature of PDIE modulator during the daytime (10:00–14:00). h) Net cooling power of the PDIE modulator under various heat transfer coefficient (*h*_c_) settings in the daytime under solar irradiation.

The PDIE modulator can be charged by UV radiation in sunlight (high emissivity state) and discharged by O_2_ in air (low emissivity state), rendering it suitable for use in the dynamic radiation cooling (DRC) field. The ideal DRC materials should exhibit low solar spectral absorption and high infrared emissivity during the daytime to realize radiative cooling (**Figure** **4**a). In addition, the materials should change from high to low infrared emissivity state at nighttime to achieve heating (Figure 4a), thereby overcoming nighttime radiative overcooling. Radiative overcooling will result in an increase in building energy consumption.^[^ ^40^ ^]^ Furthermore, the nighttime overcooling can contribute to an amplification in diurnal temperature variations of buildings, thereby jeopardizing the service life of building envelopes due to significant thermal stresses induced by enlarged temperature fluctuations.^[^ ^41^ ^]^

The PDIE modulator can adaptively regulate its infrared emissivity depend on the UV radiation in sunlight and atmospheric O_2_ without consuming energy, thus effectively alleviating nighttime radiative overcooling. This DRC performance of the PDIE modulator benefits from the high vis–NIR transmittance and infrared emissivity modulation capabilities offered by AZO NC film. The high vis–NIR transmittance enables the PDIE modulator to exhibit the solar spectrum reflection characteristics of the underlying Ag film during the daytime; the infrared emissivity modulation capabilities allow the PDIE modulator to dynamically regulate the emissivity during the daytime and nighttime. We demonstrated the DRC application of the PDIE modulator for building roofs (Figure 4b). In the daytime, the PDIE modulator reflects most of the sunlight while maintaining a high‐emissivity state, allowing the building to efficiently dissipate heat. In the nighttime, the modulator transitions to a low‐emissivity state, reducing the amount of energy radiated outward and facilitating heating within the building.

Furthermore, we investigated the DRC effect of PDIE modulator with the experimental setup shown in Figure 4c and Figure S10 (Supporting Information) (Note S4, Supporting Information). The PDIE modulator was placed on the support sticks in a polystyrene foam chamber to suppress the conductive heat gain from the back surface and side walls. The chamber was sealed using a low‐density polyethylene (PE) film as a convection shield that allows for the propagation of sunlight and infrared radiation.^[^ ^30^ ^]^ An Ag film‐deposited Al sheet and a TiO_2_‐coated Al sheet were placed in the same setup as contrast samples. Although the Ag film and the PDIE modulator exhibit similar solar spectral reflectance for reflecting sunlight, the constant low emissivity state of the Ag film (0.06 in MWIR, 0.07 in LWIR) causes it to maintain higher temperatures during the daytime and nighttime (Figure S11, Supporting Information). During the daytime (10:00–14:00), when the chamber temperature rises to an average temperature of 35.5 °C, the PDIE modulators achieve an average temperature drop of 15.1 °C compared to the Ag film‐deposited Al sheet due to the significant radiative cooling effect of PDIE modulators (Figure 4d). The PDIE modulator demonstrated superior daytime radiative cooling performance than the TiO_2_‐coated Al sheet, with an average temperature 3.33 °C lower than that of TiO_2_‐coated Al sheet. This can be attributed primarily to the slightly higher solar spectral reflectance of the PDIE modulator in the Vis‐NIR band compared to commercial TiO_2_ radiative cooling (93.1%, Figure 2b).

During the nighttime (0:00–4:00), when the average temperature of the chamber is 9.34 °C, the average temperature of the PDIE modulator was 1.24 °C higher than that of the TiO_2_‐coated Al sheet (Figure 4e), which benefits from the low emissivity of the PDIE modulator at nighttime, decreasing radiation heat loss. Unlike the commercial TiO_2_ radiative cooling materials, the PDIE modulator exhibited adaptive DRC performance of daytime cooling and nighttime heating, which can effectively reduce nighttime radiative overcooling without affecting daytime radiative cooling performance. PDIE modulators are applicable in regions characterized by significant temperature daily range, such as tropical desert climates areas, continental climate areas, and plateau regions.

We also studied the radiative cooling performance of the PDIE modulator in an open environment (considering thermal convection), and the experimental setup is shown in Figure 4f. The PDIE modulators can achieve subambient radiant cooling of 2.84 °C during the daytime without a convection shield (Figure 4g). The net cooling power of the PDIE modulator during the daytime was calculated as 67.3 W m^−2^ (Figure 4h and Note S2, Supporting Information). The cooling performance of the PDIE modulator is largely affected by nonradiative heat transfer coefficient, which is determined by atmospheric humidity, wind speed, geographical location, and other factors.^[^ ^15^ ^]^ The maximum temperature drops calculated from the heat transfer coefficient (*h*_c_) of 0, 3, 6, 9, and 12 W m^−2^ K^−1^, are 17, 10, 7, 5, and 4 K, respectively (Figure 4h). In addition to being used on roofs, by using ITO film as an infrared reflection layer and glass as a substrate, transparent PDIE modulators can be fabricated for use as smart windows for dynamically controlling infrared radiation (Note S5, Supporting Information).

## 3 Conclusion

In summary, we have developed a structurally simple PDIE modulator based on photocharging/oxidative discharging. The PDIE modulator exhibits high solar spectral reflectance and large emissivity modulation amount. These attractive properties enable PDIE modulator to efficiently realize self‐adaptive daytime radiative cooling and nighttime heating without energy consumption, making it promising for application on the roofs and windows of buildings in regions with large diurnal temperature differences. In the future, with the development of mature manufacturing processes for spraying or inkjet printing of AZO NC films, large‐scale preparation of PDIE modulator could become feasible. This would facilitate the application of PDIE modulators in buildings and vehicles to reduce energy consumption, thereby contributing to alleviating urban heat island effects and global warming.

## 4 Experimental Section

### Materials

Zinc acetate dihydrate (Zn(CH_3_COO)_2_·2H_2_O, 99%), oleylamine (OLA, 70%), and oleic acid (OA, 90%) were purchased from Sigma‐Aldrich. Aluminum acetylacetonate [Al(Acac)_3_, 99%] and oleyl alcohol (80%−85%) were obtained from Alfa Aesar. Toluene was purchased from sinopharm. An Ag pellet (99.99%) was purchased from ZhongNuo as an evaporation material. Al sheets and polyethylene terephthalate (PET) sheets were purchased from Wenghou Materials Co., Ltd.

### Synthesis of AZO NCs

The synthesis of AZO NCs is based on a slight modifications of the method published by Wainer.^[^ ^42^ ^]^ In a typical synthesis, 2.376 mmol of Zn(CH_3_COO)_2_·2H_2_O, 0.024 mmol of Al(Acac)_3_), and 2.4 mL of oleic acid were magnetically stirred under vacuum at 110 °C for 20 min. Then, 2.4 mL of OLA was injected. The precursors were further stirred under vacuum at 100 °C for 5 min and then kept at this temperature under N_2_ atmosphere for further use. Meantime, 16 mL of oleyl alcohol was heated to 280 °C under a flowing nitrogen atmosphere. Then, 2.4 mL of the precursor solution was slowly injected into hot oleyl alcohol at a rate of 0.15 mL min^−1^. After injection, the precursor was reacted for 1 h. Then, the reaction mixture was cooled to room temperature in an N_2_ atmosphere. 20 mL of ethanol was added to the reaction mixture, and the AZO NCs were separated after centrifugation at 9000 rpm for 5 min. The AZO NCs were dispersed in toluene. After two cycles of dispersion in toluene (2 mL) and precipitation by ethanol, the AZO NCs were finally dispersed in 1 mL of toluene.

### Preparation of PDIE Modulator

Ag films (≈135 nm) were deposited on the substrate materials (Al sheets, PET film) using MEB‐600 high‐vacuum electron beam evaporation system (Beijing Chuangshiweina Technology Co., Ltd., China) under 1.6 × 10^–5^ Pa pressure at 25 °C and a deposition rate of 1 Å s^−1^.

Spin‐coating was used to prepare AZO NC film on the Ag film. AZO NC suspensions (≈50 mg mL^−1^) were spin‐coated onto the samples with an initial 1000 rpm speed for 60 s followed by 4000 rpm for 20 s. Several spin coats were performed to increase the thickness of the AZO NC film. Then, ligand exchange was performed by immersing the AZO NC film in a 1 m formic acid/acetonitrile solution for 45 min. The PDIE modulator was prepared according to the steps outlined above.

### Optical Characterization

The UV energy of the solar spectrum was measured using a UV irradiance meter (SDR365, SPEEDRE Co., Ltd.) with a detection range of 320–400 nm. The spectral reflectance of the PDIE modulator was determined separately in the UV–vis–NIR (0.3–2.5 µm) and mid‐infrared (2.5–25 µm) wavelength ranges. The UV–vis–NIR reflectance spectra of PDIE modulator were measured using a UV–vis–NIR spectrophotometer (PerkinElmer LAMBDA1050) equipped with a diffuse integrating sphere. BaSO_4_ was used as standard whiteboard. The angular absolute reflectance spectra of the PDIE modulator at 0.3–2.5 µm were measured by the UV–vis–NIR spectrophotometer with a URA accessory (PerkinElmer). The infrared reflectance spectra of the PDIE modulator (2.5–25 µm) were measured using an FTIR spectrometer (Bruker Vertex 70) equipped with a mid‐infrared integrating sphere (A562, Bruker), which featured a sample port with a diameter of ≈2.1 cm and referenced to a diffuse gold standard. The infrared beam was used to illuminate the sample with an incidence angle of 13°. The diffusely reflected part of the infrared radiation was scattered inside the sphere and detected as the reflected luminous flux after integration by a detector located behind the outlet port. The angular reflectance spectra of the PDIE modulator at 2.5–25 µm were measured using the FTIR spectrometer with a variable angle reflection accessory (A513, Bruker). In all cases, the measurements were performed at ≈20 °C and the relative humidity was maintained at ≈45%.

### Morphology and Composition Analysis

The morphology of the AZO NCs was analyzed by transmission electron microscope (TEM; JEOL JEM‐2100F) operated at 200 kV. The elemental content of Al in AZO NCs was measured using induced coupled plasma optical emission spectrometer (ICP‐OES, Agilent 720ES). The AZO NCs were digested in concentrated HCl for this measurement.

The surfaces of PDIE modulators were analyzed by field‐emission scanning electron microscope (FESEM; TESCAN MIRA) with a beam energy of 20 keV. The thicknesses of AZO NC layers were measured with a Bruker Dektak XT Profiler. Cross‐sections of the PDIE modulators were prepared using a focused ion beam (FIB; FEI STRATA 400S) and then analyzed using TEM (FEI Talos F200s).

### Electron Paramagnetic Resonance Analysis

EPR measurements were collected using a Bruker EMX X‐band spectrometer with an SHQE resonator operating at 9.8 GHz. A toluene suspension of AZO NCs was sucked into a quartz capillary tube with a diameter of 1 mm. A 365‐nm UV lamp (Bruker) was used as the light source. In situ EPR measurements were performed on samples in the dark, under UV light, and several minutes after UV light exposure.

### Optical and Infrared Thermal Image Analysis

The samples were photographed using a Nikon D3100 digital camera. The samples were placed on a hot plate at 40 °C. Infrared thermal images were recorded using an FLIR T1050sc camera with a working range of 7.5–14 µm. The predefined emissivity was set to 0.95 with a focal length of 36 mm and F number of 1.15. FLIR ResearchIR Max 4.0 software was used to analyze the LWIR images. The apparent temperature of the samples was extracted after averaging using the box measurement tool in the FLIR software packages. The measurements were performed at ambient temperature of ≈20 °C and the relative humidity was maintained at ≈45%.

## Conflict of Interest

The authors declare no conflict of interest.

## Supporting information

Supporting Information

Supplemental Movie 1

## Data Availability

The data that support the findings of this study are available in the Supporting Information of this article.
