# Supplementary material for: Realizing Sunlight‐Induced Efficiently Dynamic Infrared Emissivity Modulation Based on Aluminum‐Doped zinc Oxide Nanocrystals
Source: Adv Sci (Weinh). 2024 Jul 29;11(36):2405962. doi: 10.1002/advs.202405962 (PMC11423185; doi:10.1002/advs.202405962)
Supplement: Supplementary file 1 — Supporting Information [file ADVS-11-2405962-s001.docx]

Supporting Information

Realizing sunlight-induced efficiently dynamic infrared emissivity modulation based on aluminum-doped zinc oxide nanocrystals

*Yan Jia^1^, Dongqing Liu^1^*********, Desui Chen^2^, Yizheng Jin^2^, Yufei Ge^1^, Wenxia Zhang^1^, Chen Chen^1^, Baizhang Cheng^1^, Xinfei Wang^1^, Tianwen Liu^1^, Mingyang Li^1^, Mei Zu^1^, Zi Wang^1^, Haifeng Cheng^1^*

**The** **Supplementary Materials file includes:**

Supplementary Note 1 to Note 5

Figures. S1 to S14

Table S1

Movie S1

**Supplementary Note 1: Calculation of solar reflectance and infrared emissivity.**

The solar reflectance (*R*_solar_) is calculated as the ratio of the reflected solar intensity across the solar spectrum to the integrated solar intensity, as shown below:

$R_{\text{solar}}=\frac{\int_{0}^{\infty}I_{\text{solar}}(\lambda)R(\lambda)d\lambda}{\int_{0}^{\infty}I_{\text{solar}}(\lambda)d\lambda}$ (S1)

where *I*_solar_(*λ*) represents the ASTM G173-03 global solar intensity spectrum, 𝑅(*λ*) is the spectral reflectance of the tested sample, and *λ* is the wavelength.

Since the infrared (IR) transmittance of PDIE modulator is 0,

$\alpha=1-R$ (S2)

Here, *α* and *R* are the IR absorptivity and IR reflectance of the PDIE modulator, respectively.

According to Kirchhoff’s law,

$\alpha_{\lambda}=\varepsilon_{\lambda}$ (S3)

Here, *ε_λ_* is the emissivity and *α_λ_* is the absorptivity at *λ*.

According to Equations (S2) and (S3), the emissivity of the PDIE modulator (*ε_λ_*) at wavelength *λ* can be calculated as

$\varepsilon_{\lambda}=1-R_{\lambda}$ (S4)

The band emissivity of PDIE modulator was calculated by integrating their total spectral emissivity over the blackbody radiation spectral range:

$\varepsilon\left(\lambda_{1},\lambda_{2}\right)=\frac{\int_{\lambda_{1}}^{\lambda_{2}} I_{BB}(T,\lambda)\varepsilon(T,\lambda)d\lambda}{\int_{\lambda_{1}}^{\lambda_{2}} I_{BB}(T,\lambda)d\lambda}$ (S5)

where (*λ*_1_, *λ*_2_) is the bandwidth. *ε*(*λ*_1_, *λ*_2_) is band emissivity between *λ*_1_ to *λ*_2_. *ε*(*T*, *λ*) is the emissivity of PDIE modulator at the wavelength of *λ* and temperature of *T*. *I_BB_* (*T*, *λ*) is the spectral intensity emitted by the blackbody at the wavelength of *λ* and temperature of *T*.

**Supplementary Note 2: Calculations of the infrared radiation power and cooling power of PDIE modulators.**

The IR radiation power of the PDIE modulator at temperature *T* was calculated as follows:

$P\left(\lambda_{1},\lambda_{2}\right)=\varepsilon\left(\lambda_{1},\lambda_{2}\right)\times\int_{\lambda_{1}}^{\lambda_{2}} I_{BB}(T,\lambda)d\lambda$ (S6)

The change in the IR radiation power of the PDIE modulator due to the change in the emissivity state was calculates as follows:

$\Delta P\left(\lambda_{1},\lambda_{2}\right)=P_{h}(\lambda_{1},\lambda_{2})-P_{l}(\lambda_{1},\lambda_{2})$ (S7)

Here, *P_h_*(*λ*_1_, *λ*_2_) and *P_l_*(*λ*_1_, *λ*_2_) represent the IR radiation powers of the PDIE modulator at high and low emissivity states, respectively.

In an open environment, the sample will emit heat through the surface, and the absorbed heat power includes the heat from solar radiation (*P*_solar_), ambient radiation (*P*_amb_) and heat transfer by conduction and convection due to temperature differences (*P*_conv + cond_). The net cooling power (*P*_cool_) refers to the difference between the radiated power and the absorbed power, expressed as:

$P_{\text{cool}}=P_{\text{rad}}-P_{\text{amb}}-P_{\text{solar}}-P_{\text{conv+cond}}$ (S8)

At night, due to the disappearance of solar radiation, the net cooling power expression can be simplified to:

$P_{\text{cool}}=P_{\text{rad}}-P_{\text{amb}}-P_{\text{conv+cond}}$ (S9)

Specifically, the radiated energy through the PDIE modulator (*P*_rad_) is:

$P_{\text{rad}}=A\int d\text{Ω}\cos\theta\int_{0}^{\infty} d\lambda I_{\text{BB}}\left(T,\lambda\right)\varepsilon(\lambda,\theta)$ (S10)

The absorbed energy from ambient radiation (*P*_amb_) is:

$P_{\text{amb}}=A\int d\text{Ω}\cos\theta\int_{0}^{\infty} d\lambda I_{\text{BB}}\left(T_{\text{amb}},\lambda\right)\varepsilon(\lambda,\theta)\varepsilon_{\text{atm}}(\lambda,\theta)$ (S11)

The absorbed energy from solar radiation (*P*_solar_) is:

$P_{\text{solar}}=\int_{0}^{\infty} d\lambda\varepsilon(\lambda,\theta)I_{\text{solar}}\left(\lambda\right)$ (S12)

The lost energy due to convection and conduction (*P*_conv + cond_) is:

$P_{\text{conv+cond}}(T_{\text{s}},T_{\text{amb}})=Ah_{\text{c}}(T_{\text{amb}}-T_{\text{s}})$ (S13)

where *A* is the radiation area, 𝜃 is the local zenith angle, *T*_amb_ is the ambient temperature, *ε*_atm_(*λ*, 𝜃) is related to atmospheric transmittance, wavelength, and zenith angle, which can be obtained according to *ε*_atm_(*λ*, 𝜃) = 1–t(*λ*)^1/cos𝜃^, where t(*λ*) is the atmospheric transmittance at zenith angle 𝜃. *T*_s_ is the temperature covered by the sample and *T*_amb_ is ambient temperature. Typical nonradiative heat transfer coefficient *h*_c_ is in the range of 2–12 W·m^−2^·K^−1^.

**Supplementary Note 3: Electromagnetic power loss density Simulation.**

The dielectric function of AZO NCs with high and low carrier concentrations (4.7×10^19^ cm^-3^ and 4.03×10^19^ cm^-3^) was calculated using the Drude-Lorentz model (Equations (S14) and (S15)) in *Matlab*. *ε*_p_(*ω*) is dielectric function of AZO NCs, *ε*_∞_ is the high-frequency dielectric constant, *ω*_p_ is the bulk plasma frequency of free carriers, *γ* is bulk collision frequency, *n* is free carrier concentration, *ε*_0_ is free space permittivity, and *m*_e_ is electron effective mass.

A 14 nm spherical model is set up in *COMSOL Multiphysics*, and the dielectric function of AZO NCs is used as the material parameter for modeling. Utilizing Wave Optics Module of *COMSOL Multiphysics*, the surface and multi-faceted electromagnetic power loss density of spherical model is calculated.

$\varepsilon_{\text{p}}(\omega)=\varepsilon_{\infty}-\frac{\omega_{\text{p}}^{2}}{\omega^{2}-i\omega\gamma}$ (S14)

$\omega_{\text{p}}=\sqrt{\frac{ne^{2}}{\varepsilon_{0}m_{\text{e}}}}$ (S15)

**Supplementary Note 4: Demonstration of dynamic radiative cooling performance of PDIE modulators.**

To investigate the dynamic radiative cooling performance of PDIE modulators, on-site measurements were carried out using a self-designed cooling apparatuses for PDIE modulator on March 5th, 2023, in Changsha, China. Ag film coated Al sheet and TiO_2_-coated Al sheet were used as reference samples. Figure 4c of the main text and Figure S8 shows the setup. A chamber was carved into a polystyrene foam box, which was then wrapped with Al foil. A thermocouple was glued on the back of the samples using Al foil with back glue to monitor the temperature of the samples. A thermocouple was dangled inside the chamber to monitor the chamber temperature. The chamber was sealed using a PE film to allow the transmission of the solar spectrum while avoiding thermal convection between the sample and the surrounding environment. The reference samples were placed in the same apparatus and measured alongside the test sample. To measure the total solar intensity (including direct and diffuse reflection), a pyranometer (Susong, TBQ-B) was placed next to the samples and connected to a computer.

We also studied the daytime radiative cooling properties of samples without the convection shield that allows for thermal convection. The schematic diagram of this setup is shown in Figure 4f. The temperature fluctuations of the sample and environment were significant in this case due to the convective influence of airflows.

**Supplementary Note 5: The preparation and properties of transparent PDIE modulators.**

By using ITO film as an infrared reflection layer and glass as a substrate, transparent PDIE modulators can be fabricated for use as smart windows in the field of energy-saving architecture (Figure S10). The PDIE modulators can independently and adaptively regulate infrared emissivity depend on sunlight while maintaining 88.3% visible light transparency (Figure S11). The emissivity modulation amounts of transparent PDIE modulators at MWIR and LWIR are 0.32 and 0.45 respectively, which is slightly lower than that of devices with Ag films as infrared reflective layers. This is because the infrared reflectivity of ITO film (77.2% in 3–13 μm) is lower than that of Ag film (97.7% in 3–13 μm; Figure S12), which reduces the infrared emissivity modulation amount of transparent PDIE modulators.

**

**

Figure S1 XRD pattern of AZO NC film.





Figure S2 LSPR peak of AZO NC film (peak position: 7.09 μm).





Figure S3 Visible and near-infrared (Vis-NIR) transmittance of an AZO NC film (~1.4 μm).


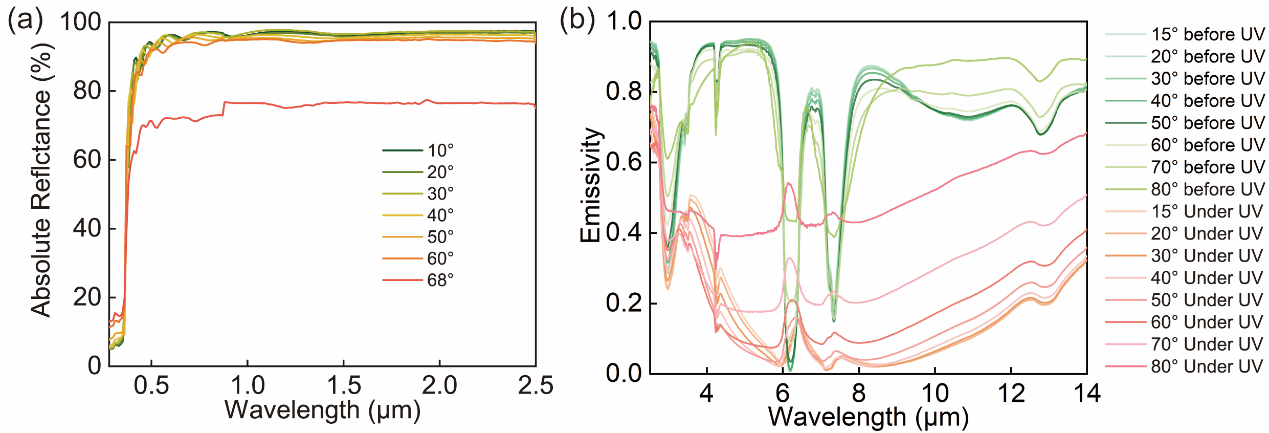


Figure S4 (a) The absolute reflectance of PDIE modulator at 10° to 68°. (b) The emissivity change of PDIE modulator at 15° to 80° before and under UV.





Figure S5 Temperature change of the PDIE modulator (measured by thermocouples) during UV irradiation (irradiance: 5 mW/cm^2^) with the sample placed on a 40 °C hot plate.


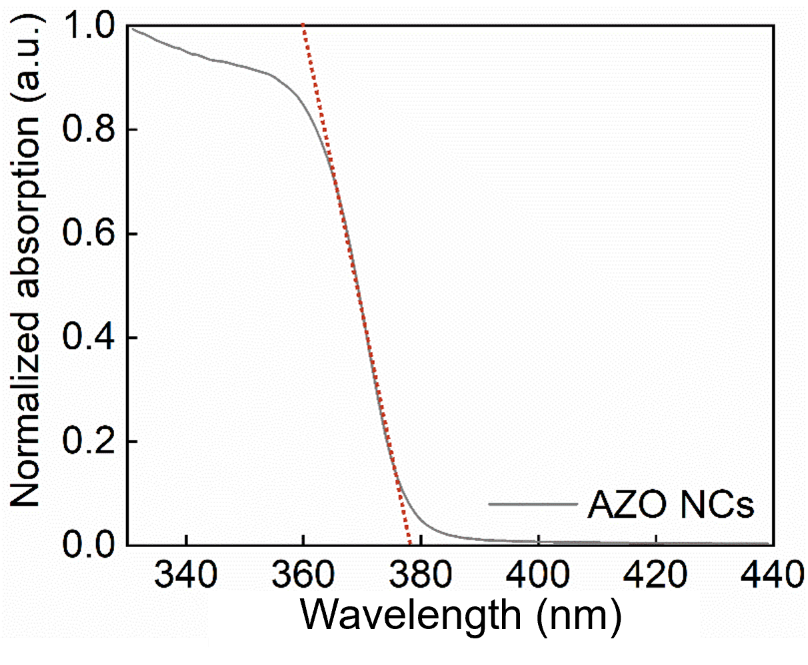


Figure S6 Optical cutoff wavelength of AZO NCs.





Figure S7 UV irradiance of sunlight (March 06, 2023, in Changsha, China).


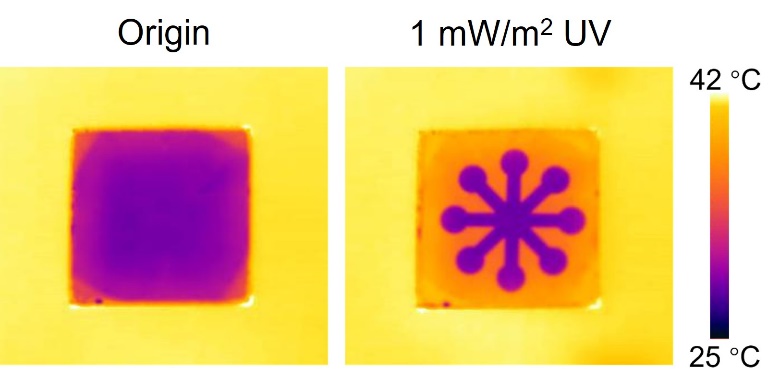


Figure S8 Infrared images of the PDIE modulator before and after exposure to 1 mW/cm^2^ UV light with UV mask. The PDIE modulator was placed on a hot plate at 40 °C.


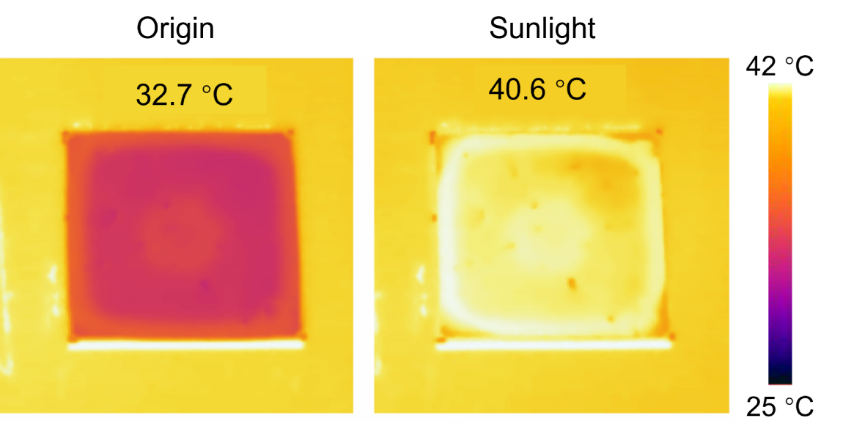


Figure S9 Infrared images of the PDIE modulator before and after exposure to sunlight with 1.92 mW/cm^2^ UV irradiance. The PDIE modulator was placed on a hot plate at 40 °C.


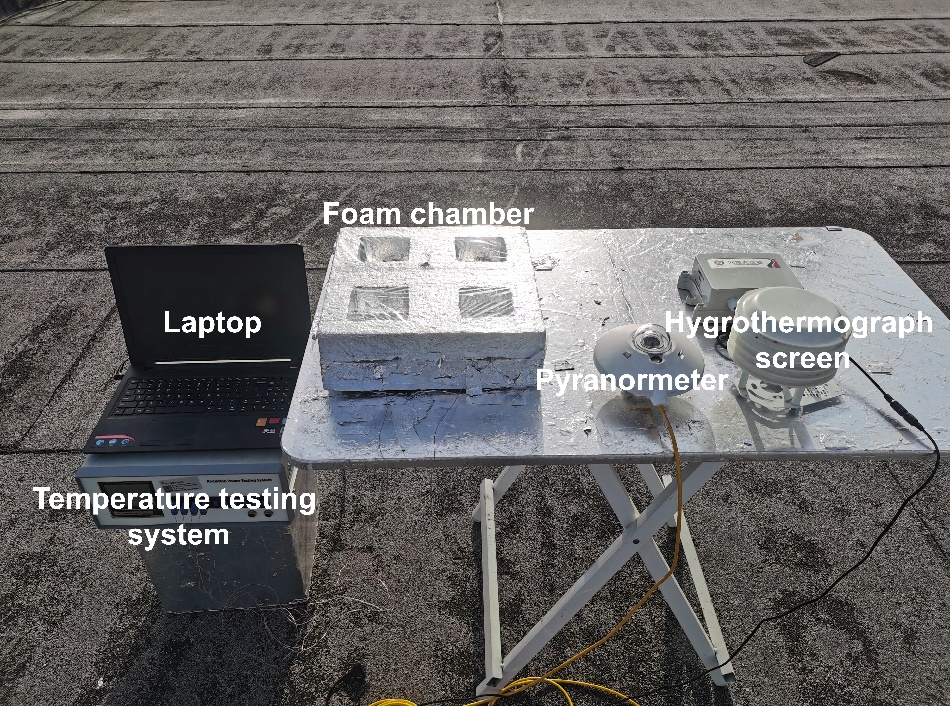


Figure S10 Photograph of the setup used for testing the dynamic radiation cooling performance of PDIE modulators.


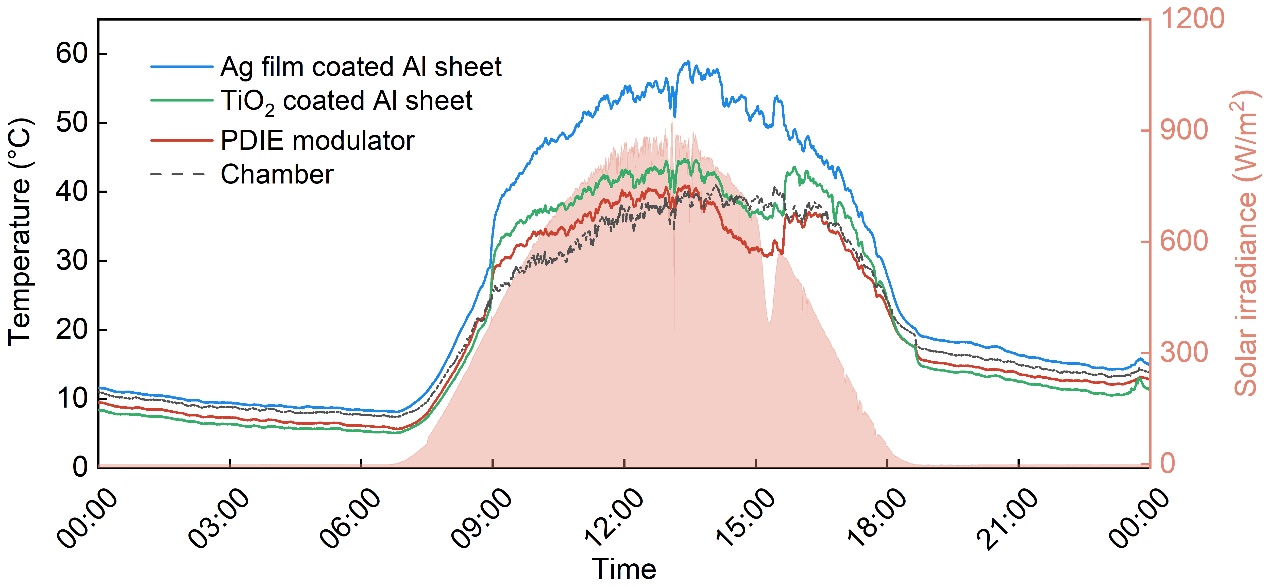


Figure S11. Real-time all-day solar radiation intensity and temperatures of a PDIE modulator applied the experimental setup shown in Figure 4c and Figure S7. The temperatures of Ag film coated Al sheet and commercial TiO_2_ coated Al sheet (film thickness: ≈250 μm) were also recorded as reference samples. At 14:00-15:30, the temperature of the samples decreased because the samples were in a shaded area and did not receive direct solar radiation. Date and location: March 5th, 2023; Changsha, China (28°15′N, 112°59′E).


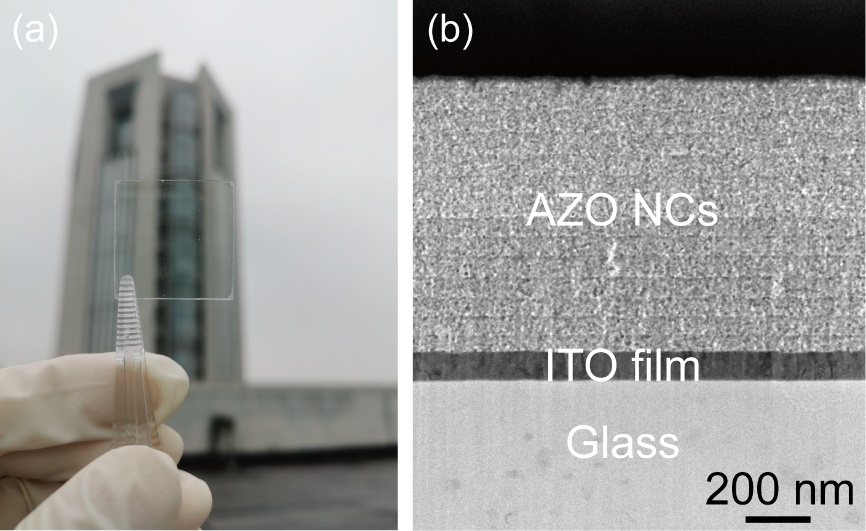


Figure S12 (a) Optical photo of the transparent PDIE modulator. (b) Cross-sectional morphologies of the transparent PDIE modulator.





Figure S13 Visible transmittance and infrared emissivity spectra of the transparent PDIE modulator at nighttime and daytime with sunlight.





Figure S14 Infrared reflectance spectra of Ag film and ITO film.

Table S1 Comparison of different infrared emissivity regulation devices.

| **Work mechanism** | **Materials** | **Infrared emissivity regulation** | | **Response time** | **Cycling stability** | **Spectral range** | **Ref.** |
| --- | --- | --- | --- | --- | --- | --- | --- |
|  |  | **MWIR** | **LWIR** |  |  |  |  |
| temperature-induced | GST | - | 0.324 | Several  seconds | - | 7.5–13 μm | ^[1]^ |
|  |  | - | ~0.7 | ~60 s | - | 7.5–13 μm | ^[2]^ |
|  | VO_2_ | - | 0.37 | 0.04 s | 10^5^ | 7.5–13 μm | ^[3]^ |
|  |  | - | 0.26 | - | - | 1.6-20 μm | ^[4]^ |
|  |  | - | 0.4 | - | - | 2.5–15 μm | ^[5]^ |
|  | W*_x_*V_1-_*_x_*O_2_ | - | 0.7 | - | - | 6–16 μm | ^[6]^ |
| gas-induced | yttrium | 0.25 | 0.39 | < 40 s | >100 | 2.5–15 μm | ^[7]^ |
| mechanical-induced | Dielectric elastomer actuator | 0.28 | 0.27 | <1 s | >750 | 2.5–15 μm | ^[8]^ |
|  | Bioinspired composite material | - | 0.33 | - | - | 5–16 μm | ^[9]^ |
|  | Reconfigurable graphene device | 0 | 0.265 | - | >30 | 7.5–15 μm | ^[10]^ |
| electro-induced | Solid-state electrochromic | 0.52 | 0.78 | ≥4 min | - | 2–26 μm | ^[11]^ |
|  | WO_3_ | 0.727 | 0.153 | 180 s | - | 2.5–13 μm | ^[12]^ |
|  | Li_4_Ti_5_O_12_ | 0.68 | 0.315 | 1 min | 100 | Visible and infrared | ^[13]^ |
|  | PANI | 0.11 | 0.436 | <2 s | 500 | 2.5–18 μm | ^[14]^ |
|  | PANI | 0.183 | 0.388 | - | - | 2.5–25 μm | ^[15]^ |
|  | Graphene | - | 0.45 | 1 s | 2200 | Visible, infrared, and microwave | ^[16]^ |
|  | Multiwalled carbon nanotubes | - | 0.7 | 1 s | 3500 | 7.5–13 μm | ^[17]^ |
|  | Ag | 0.77 | 0.71 | ≤15 s | ≥350 | Visible, infrared | ^[18]^ |
| electro-induced | Cu | - | 0.85 | - | 2500 | Visible, infrared | ^[19]^ |
|  | Quantum wells |  | 0.5 | 10 kHz | - | 9.1–10 μm | ^[20]^ |
|  | Graphene resonator | 0.02 |  | 2 kHz | - | 6.25–8.3 μm | ^[21]^ |
| photo-induced | Metamaterials | - | 0.12 | 33.7 s | - | 8–16 μm | ^[22]^ |
|  | GST | - | 0.6 | - | - | 2.5–15 μm | ^[23]^ |
|  | GST | - | 0.23 | - | - | 2.5–15 μm | ^[24]^ |
|  | VO_2_ | 0.8 | 0.41 | - | 100 | 3–14 μm | ^[25]^ |
|  | GST | - | 0.62 | - | - | 2.5–15 μm | ^[26]^ |
|  | **AZO NCs** | **0.72** | **0.61** | **16.3 s** | **>500** | **2.5–15 μm** | **This work** |

[1] Y. Qu, Q. Li, K. Du, L. Cai, J. Lu, M. Qiu, *Laser & Photonics Reviews*, **2017**, 11, 1700091.

[2] Y. Qu, Q. Li, L. Cai, M. Pan, P. Ghosh, K. Du, M. Qiu, *Light: Science & Applications* **2018**, 7, 26.

[3] L. Xiao, H. Ma, J. Liu, W. Zhao, Y. Jia, Q. Zhao, K. Liu, Y. Wu, Y. Wei, S. Fan, K. Jiang, *Nano Letters* **2015**, 15, 8365.

[4] K. Sun, W. Xiao, C. Wheeler, M. Simeoni, A. Urbani, M. Gaspari, S. Mengali, C. H. d. Groot, O. L. Muskens, *Nanophotonics* **2022**, 11, 4101.

[5] S. Wang, T. Jiang, Y. Meng, R. Yang, G. Tan, Y. Long, *Science* **2021**, 374, 1501.

[6] K. Tang, K. Dong, J. Li, M. P. Gordon, F. G. Reichertz, H. Kim, Y. Rho, Q. Wang, C.-Y. Lin, C. P. Grigoropoulos, A. Javey, J. J. Urban, J. Yao, R. Levinson, J. Wu, *Science* **2021**, 374, 1504.

[7] B. Cheng, D. Liu, Y. Jia, H. Cheng, *Advanced Optical Materials* **2022**, 10, 2201702.

[8] C. Xu, G. T. Stiubianu, A. A. Gorodetsky, *Science* **2018**, 359, 1495.

[9] E. M. Leung, M. Colorado Escobar, G. T. Stiubianu, S. R. Jim, A. L. Vyatskikh, Z. Feng, N. Garner, P. Patel, K. L. Naughton, M. Follador, E. Karshalev, M. D. Trexler, A. A. Gorodetsky, *Nature Communications* **2019**, 10, 1947.

[10] A. Krishna, J. M. Kim, J. Leem, M. C. Wang, S. Nam, J. Lee, *Nano Letters* **2019**, 19, 5086.

[11] H. Demiryont, D. Moorehead, *Solar Energy Materials and Solar Cells* **2009**, 93, 2075.

[12] K. Sauvet, L. Sauques, A. Rougier, *Solar Energy Materials and Solar Cells* **2009**, 93, 2045.

[13] J. Mandal, S. Du, M. Dontigny, K. Zaghib, N. Yu, Y. Yang, *Advanced Functional Materials* **2018**, 28, 1802180.

[14] P. Chandrasekhar, B. J. Zay, G. C. Birur, S. Rawal, E. A. Pierson, L. Kauder, T. Swanson, *Advanced Functional Materials* **2002**, 12, 95.

[15] Y. Tian, X. Zhang, S. Dou, L. Zhang, H. Zhang, H. Lv, L. Wang, J. Zhao, Y. Li, *Solar Energy Materials and Solar Cells* **2017**, 170, 120.

[16] M. S. Ergoktas, G. Bakan, E. Kovalska, L. W. Le Fevre, R. P. Fields, P. Steiner, X. Yu, O. Salihoglu, S. Balci, V. I. Fal’ko, K. S. Novoselov, R. A. W. Dryfe, C. Kocabas, *Nature Photonics* **2021**, 15, 493.

[17] Y. Sun, H. Chang, J. Hu, Y. Wang, Y. Weng, C. Zhang, S. Niu, L. Cao, Z. Chen, N. Guo, J. Liu, J. Chi, G. Li, L. Xiao, *Advanced Optical Materials* **2020**, 9, 2001216.

[18] M. Li, D. Liu, H. Cheng, L. Peng, M. Zu, *Science Advances* **2020**, 6, eaba3494.

[19] C. Sui, J. Pu, T.-H. Chen, J. Liang, Y.-T. Lai, Y. Rao, R. Wu, Y. Han, K. Wang, X. Li, V. Viswanathan, P.-C. Hsu, *Nature Sustainability* **2023**, 6, 428.

[20] T. Inoue, M. D. Zoysa, T. Asano, S. Noda, *Nature Materials* **2014**, 13, 928.

[21] V. W. Brar, M. C. Sherrott, M. S. Jang, S. Kim, L. Kim, M. Choi, L. A. Sweatlock, H. A. Atwater, *Nature Communications* **2015**, 6, 7032.

[22] Z. J. Coppens, J. G. Valentine, *Advanced Materials* **2017**, 29, 1701275.

[23] Z. Xu, H. Luo, H. Zhu, Y. Hong, W. Shen, J. Ding, S. Kaur, P. Ghosh, M. Qiu, Q. Li, *Nano Letters* **2021**, 21, 5269.

[24] C. Kim, Y. Kim, M. Lee, *Advanced Materials Technologies* **2022**, 7, 2101349.

[25] X. Jiang, X. Wang, J. Nong, G. Zhu, X. He, T. Du, H. Ma, Z. Zhang, H. Chen, Y. Yu, *ACS Photonics* **2024**, 11, 218.

[26] D. Kang, Y. Kim, M. Lee, *ACS Applied Materials & Interfaces* **2024**, 16, 4925.
